# Small, medium, large or supersize? The development and evaluation of interventions targeted at portion size

**DOI:** 10.1038/ijo.2014.84

**Published:** 2014-07-25

**Authors:** W M Vermeer, I H M Steenhuis, M P Poelman

**Affiliations:** 1 Department of Gynecology, Leiden University Medical Centre, Leiden, The Netherlands; 2 Department of Health Sciences and the EMGO Institute for Health and Care Research, Faculty of Earth and Life Sciences, VU University Amsterdam, Amsterdam, The Netherlands

## Abstract

In the past decades, portion sizes of high-caloric foods and drinks have increased and can be considered an important environmental obesogenic factor. This paper describes a research project in which the feasibility and effectiveness of environmental interventions targeted at portion size was evaluated. The studies that we conducted revealed that portion size labeling, offering a larger variety of portion sizes, and proportional pricing (that is, a comparable price per unit regardless of the size) were considered feasible to implement according to both consumers and point-of-purchase representatives. Studies into the effectiveness of these interventions demonstrated that the impact of portion size labeling on the (intended) consumption of soft drinks was, at most, modest. Furthermore, the introduction of smaller portion sizes of hot meals in worksite cafeterias in addition to the existing size stimulated a moderate number of consumers to replace their large meals by a small meal. Elaborating on these findings, we advocate further research into communication and marketing strategies related to portion size interventions; the development of environmental portion size interventions as well as educational interventions that improve people's ability to deal with a ‘super-sized' environment; the implementation of regulation with respect to portion size labeling, and the use of nudges to stimulate consumers to select healthier portion sizes.

## Introduction

In the 1970s, fast-food restaurant marketing directors conceived that consumers would buy more of their products if they were sold in large sizes and if value size pricing (that is, a lower price per unit for large portion sizes compared to small portion sizes) was employed.^1^ Rapidly, other companies in the fast food and packaged-food industry copied the strategy of increasing portion sizes and value size pricing, and a culture of supersizing was steadily created.^2,3^

Several studies show that portion sizes, especially of high-caloric foods and drinks, have increased enormously in recent decades.^4, 5, 6, 7, 8^ Although the United States is known for its supersizing practices, portion sizes have also increased in Europe.^4,6^ Barbara Rolls has illustrated in numerous studies (described elsewhere in this supplement) that large portion sizes of (energy-dense) food may lead to an increased food intake. Many studies have found that large portion sizes enhance consumption both in the case of single-meal settings^9, 10, 11^ and in cases when people are chronically exposed to large portion sizes.^12,13^

In combination with the development of growing portion sizes, norms about standard quantities that are appropriate to consume have gradually changed and many people have difficulties defining what a normal portion size is. This phenomenon has been described as ‘portion distortion' and studies have found that in the case of high-caloric foods and drinks, people tend to perceive larger quantities than recommended by the dietary guidelines, as appropriate amounts to eat at a single occasion.^14, 15, 16^

Supersized portions of high-caloric foods and drinks can be considered the key environmental obesogenic factors. The ANalysis Grid for Environments Linked to Obesity is a framework that has been developed for identifying and prioritizing environmental interventions for obesity).^17^ Within this framework, large portion sizes combined with value size pricing are situated in the physical and economic environment and can be considered a valid starting point for the development of environmental interventions directed at the prevention of weight gain. This paper describes a research project that we conducted aiming to evaluate the feasibility and effectiveness of environmental interventions targeted at portion size. The studies that were part of this project have been published in more detail elsewhere.^18, 19, 20, 21, 22, 23, 24, 25^ Therefore, only a short overview of the main findings and the references will be given in this paper. This paper will focus on putting the results of our research project in a broader perspective and providing future directions for both research and practice.

## The development and feasibility of interventions targeted at portion size

**Figure 1:**
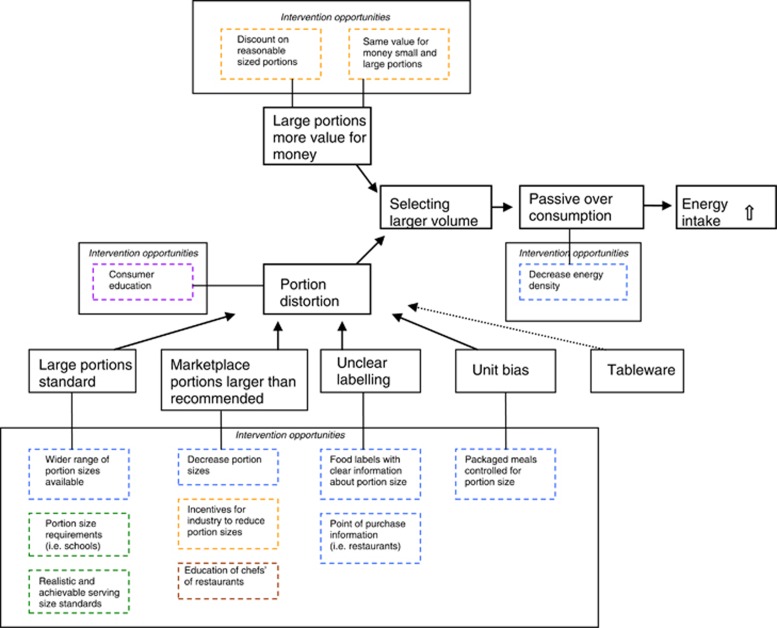
Framework for portion size interventions. Original work published under CC-BY 2.0 license in *International Journal of Behavioral Nutrition and Physical Activity* 2009, 6:58, doi:10.1186/1479-5868-6-58, 2009 Steenhuis and Vermeer; licensee BioMed Central Ltd.

The onset of the research project consisted of conducting a review identifying possibilities for interventions targeted at portion size. On the basis of the intervention studies that were reviewed, we developed a framework for portion size interventions,^18^ see Figure 1. The framework shows that the underlying factors causing portion distortion can be diminished by environmental interventions in, among others, the physical and economic food environment. Furthermore, interventions that are targeted at the selection of food are of great value because once a larger portion is selected, over-consumption is very likely to occur. On the basis of the literature review the following interventions were identified to study further: offering a larger variety of small portion sizes, reducing portion sizes, pricing strategies such as proportional pricing (that is, removing beneficial prices for large sizes by keeping the price per gram stable along different sizes), and portion size labeling.

After identifying opportunities for interventions targeted at portion size, their feasibility was assessed in two qualitative studies. The perspectives of both consumers^25^ and point-of-purchase representatives were taken into account.^24^ On the basis of the attitudes that the study participants of both studies expressed towards various interventions targeted at portion size, we concluded that adding smaller sizes to the assortment and portion size labeling were the most feasible interventions to implement. In settings such as worksite cafeterias, a proportional pricing strategy was considered to be feasible as well. After having identified these three environmental interventions as feasible, a number of studies were conducted in order to evaluate to which extent they were effective in altering consumers' food intake.

### The effectiveness of portion size labeling

As illustrated elsewhere in this supplement by Christina Roberto, food labels can provide consumers with simple and practical information, which can empower them to make healthier choices.^26^ Our aim was to study whether portion size labeling was a helpful tool for consumers to select appropriate portion sizes. Before evaluating the impact of portion size labeling on size choices and consumption behavior, we conducted a questionnaire study in order to identify the most promising format of portion size labeling.^21^ The results showed that reference portion size labeling (that is, labels that provided consumers with a reference portion size) increased the likelihood that participants chose small sizes of soft drink compared with labels that only communicated the amount of soft drink in milliliters (although this was a statistical trend). The stimulus material in this study however was presented through photographs. In a subsequent field experiment, the effectiveness of reference portion size and caloric Guidelines for Daily Amounts labeling on consumers' portion size choices and consumption was assessed.^22^ Portion size and caloric Guidelines for Daily Amounts labeling were found to have no effect on soft drink intake. This is in line with two other studies that did not demonstrate effects of portion size labeling on size choice, consumption or purchase behavior either.^27, 28, 29^ All in all, the two studies that we conducted to assess the impact of portion size labeling did not provide unambiguous conclusions and suggest that the effects of portion size labeling changing the preferences to smaller portions are expected, at most, to be small.

### The effectiveness of a larger variety of portion sizes and proportional pricing

One of the reasons why large portions are preferentially consumed is value for money and, as mentioned above, in many settings value size pricing is employed. Conversely, pricing strategies could be used to stimulate smaller size choices by proportional pricing of small and large portions. Studies have demonstrated the effectiveness of pricing strategies targeted at altering the type of food that consumers purchase.^30,31^ Only one study was known, however, assessing the impact of (a single exposure to) pricing strategies on portion size choices.^28^ We studied the impact of a larger variety of small portion sizes and proportional pricing of hot meals in a longitudinal randomized controlled trial that took place in 26 worksite cafeterias.^23^ Participating worksite cafeterias were randomly allocated to either an experimental condition in which a smaller portion was offered in addition to the existing portion and proportional pricing was employed, an experimental condition in which a smaller portion was added to the assortment and value size pricing was employed, or the control condition in which only the existing size of the hot meal was available. The results showed that after the introduction of small meals, a small group (that is, 10%) of worksite cafeteria visitors replaced their large meals with small meals, but that proportional pricing had no effect. It is possible that proportional pricing did not affect purchase behavior because the price differences between the portion sizes were too small and the prices in worksite cafeterias are generally low. The results also showed that the sales figures of fried snacks did not increase, suggesting that consumers did not compensate for their small meals by purchasing more snacks and thereby eliminating the beneficial effects of selecting the small portion size. On the other hand, based on consumer data, there were some indications of compensatory food intake: 14% of the participants who chose a small meal in the worksite cafeteria reported, among other things, having larger meals than they usually have at home. Finally, the small meal attracted a relevant target group, as small meal purchases were positively related to being female and to body mass index (the latter was borderline significant). In addition to evaluating the effect of introducing smaller meals and proportional pricing in worksite cafeterias, a process evaluation was carried out.^20^ The results showed that offering a small meal, in addition to the existing size meal, as well as proportional pricing were generally implemented as prescribed by the protocol.

## Public health implications

The studies that we conducted revealed that portion size labeling, offering a larger variety of portion sizes, and proportional pricing were feasible interventions to implement according to both consumers and point-of-purchase representatives. With respect to the effectiveness of these interventions, our results indicated that the effect of portion size labeling on the (intended) consumption of soft drinks was, at most, modest. However, a format indicating the number of servings per soft drink cup seemed most promising. Lastly, the introduction of a smaller portion size of hot meals in worksite cafeterias in addition to the existing size could be considered a sustainable intervention that can help a reasonable and relevant proportion of guests to replace their large meal with a small meal. There is however a risk of compensatory food intake.

On the basis of the results of this research project, some public health implications and suggestions for further research will be summarized. These implications and research suggestions will be organized along the theme communication of interventions targeted at portion size, education about dealing with large portion sizes, regulation with respect to portion size labeling and nudging.

### Communication to the consumers of interventions targeted at portion size

During the focus group discussions with the consumers, some participants mentioned that the acceptability of such interventions would depend on how these interventions were to be communicated to the general public. Developing adequate communication strategies for public health interventions is generally an important but challenging aspect of the implementation process.^32^ With respect to the communication of interventions targeted at portion size there are some possibilities. A first option is to communicate the health aspects as the rationale behind these interventions. There are, however, two aspects that hamper this approach. First, our focus group study results showed that some consumers considered interventions targeted at portion size (such as the reduction of portion sizes) as paternalistic.^25^ This topic will be further discussed later in this paper. Second, for many, the connection between portion sizes and weight control is not that obvious. Often, people think that the type of food is important rather than how much of it is consumed. This is illustrated by a survey study from the American Institute for Cancer Research showing that 78% of the respondents believed that eating certain types of foods and avoiding others is more important than the amount of food that is consumed.^33^

Besides emphasizing health as the rationale behind interventions targeted at portion size, another option is to stress other aspects when communicating such interventions to consumers. The idea behind this strategy is that, in general, companies are reluctant to reduce their prices along with reducing portion sizes. On the other hand, consumers want value for their money,^1^ and are therefore likely to distrust the motives of companies that reduce their portion sizes (most likely without lowering their prices). Lowering prices along with a reduction in portion sizes is generally not that obvious. The main reason for this is that the costs of many food and drink items are mainly due to the costs of their development, marketing and the logistics behind them as opposed to costs of the commodities themselves. Again, this stresses the importance of adequately communicating interventions targeting portion size. An option for the communication of portion size reductions would therefore be to emphasize other types of value such as quality or exclusivity. An example is the pricing of soft drinks in fast-food settings compared with cafes and restaurants. Informal observations in fast-food restaurants in the Netherlands show that consumers pay approximately €0.42 per 100 ml for soft drinks that are served in disposable cups containing 300–500 ml. In regular cafes and restaurants, soft drinks are often sold in glass bottles containing 200 ml. The bottles might contribute to a more positively perceived taste, quality and exclusivity. Accordingly, consumers are prepared to pay approximately €1.10 per 100 ml for a seemingly identical product (although it should be mentioned that post mix soft drinks that are served in fast-food restaurants are somewhat differently produced than soft drinks that are sold in bottles). Obviously, this does not apply to all products and there is a large group of consumers who equate value-for-money to quantity rather than to quality. Nevertheless, it would be worthwhile to assess the effects of selling (a sense of) quality and ‘premium-ness', rather than translating value into large sizes. Social marketing experts aim to develop and deliver products that offer real value to the customer by using a combination of all the marketing components (that is, product, price, place and promotion) and their insights could be helpful for the development of positioning strategies with respect to smaller portion sizes.^34^

A last option would be not communicating portion size reductions at all. This could be achieved by a gradual and implicit or ‘stealth' reduction in portion sizes. Akin to portion sizes that have not increased overnight, reductions in portion sizes could also be carried out steadily over time. An example is single candy bars, available at different point-of-choice settings, that weighed 60 g in 1999, then reduced to 54 g in 2001 and then to 51 g in 2008 (ref. 6)a 15% decrease in less than 10 years. To our knowledge, this has not been explicitly communicated, nor were the prices reduced. As this has not led to a boycott of the manufacturer of this candy bar, in some cases not communicating portion size reductions seems to be an option. Another advantage of stealth portion size reduction is that cognitive signals triggering compensatory food intake are less likely to occur. On the other hand, as the point-of-purchase representatives mentioned, there is a risk of a loss of credibility that many companies are unwilling to take. A last stealth intervention strategy that is worth investigating is a combination of portion size reduction and a decrease in energy density (that is, the number of calories per gram). Rolls *et al.*^35^ have studied the combined effects of reductions of portion sizes and lowering the energy density and concluded that consuming low-energy-dense foods allows individuals to decrease energy intake while still consuming satisfying portions and maintaining satiety (see elsewhere in this supplement). It is however unclear which products are suitable for stealth portion size reductions, and to what extent portion size reductions (possibly accompanied by a decrease in energy density) can take place without a loss of credibility or increasing the risk of compensation behavior. In general, further research into communication and marketing strategies related to portion size interventions is needed.

### Education about dealing with large portion sizes

On the basis of the qualitative studies among both consumers and point-of-purchase representatives, the most realistic scenario with respect to implementing interventions targeted at portion sizes is that smaller portion sizes of high-caloric products are being made available *without removing* the large ones. Therefore, interventions targeted at portion size should have a multiple focus. First, interventions are needed that are situated in point-of-purchase settings, as is the case with the studies that were part of this research project. Secondly, interventions targeted at portion size could train people how to deal with a ‘super-sized' food environment by educating them about portion distortion, self-regulation and pre-meal planning.^36, 37, 38^ For instance, the PortionSize@warenessTool is aimed at addressing awareness of reference portion sizes as well as the range of factors that trigger overconsumption from larger portions.^38^ Furthermore, we (IHS, MPP) are currently evaluating a comprehensive educational portion control intervention program among overweight and obese individuals. This program aims to (1) increase portion size awareness;^38^ (2) enhance self-regulation skills regarding portion control;^39^ (3) improve portion and energy-density control cooking skills; and (4) support people creating a portion size-friendly home environment. The program is based on theoretical insights from self-regulation theory, action planning and coping planning, and is directed at improving people's ability to control and maintain adequate portion size selection and intake, thereby resisting environmental stimuli by which one is triggered to select large food portions.

### Regulation with respect to portion size labeling

Labeling generally seems to be a widely endorsed intervention in the battle against obesity. However, this approach seems to be founded on overly high expectations with respect to the impact of labeling. Following from our and others' study findings,^28,29,40^ we cannot affirm that portion size labeling has a convincing impact on consumer behavior. It is therefore concluded that transparent and correct information should lie at the basis of a healthy food environment, but that it is not sufficient to improve consumers' eating patterns. This means that high-quality information provision with respect to portion sizes is a minimal and crucial aspect of a healthy nutrition environment. Unfortunately, current portion size labeling is often not in line with marketplace portions and therefore confusing.^41^ A recent study has found that consumers are more willing to believe (and consequently consume more when confronted with) a label that depicts a medium-sized item as a smaller item than as a larger item. Thus, a small-sized item that is mislabeled as ‘large' (or ‘medium') is less likely to be believed than a large-sized item that is mislabeled as ‘small' (or ‘medium'). Consumers are not aware of this phenomenon that has been described as the ‘asymmetric size label effect'.^42^ This means that due to the asymmetric size label effect, consumers could be more likely to believe the portion size information from manufacturers (suggesting large reference sizes) than based on nutritional guidelines (suggesting small reference portion sizes).

All in all, communication with respect to (reference) portion sizes is currently ambiguous and therefore often not helping consumers in making informed choices. Another problem is that the information provided is often not in line with the nutritional guidelines. We therefore advocate regulations that enforce clear and realistic norms with respect to reference portion sizes and their communication and labeling.

### Nudging

Over time, portion sizes have gradually and ‘stealthily' increased, and thereby contributed to the prevalence of overweight and obesity.^43^ An important strength of our studies is that we evaluated interventions that were judged by stakeholders to be feasible to implement (that is, portion size labeling, proportional pricing and adding smaller sizes to the portfolio). However, the study results demonstrated only modest effects of the feasible interventions on behavior.

A (gradual) decrease in portion sizes (as mentioned above) and an elimination of the largest portion sizes of high-caloric snacks and drinks would be more invasive interventions hampering consumers to select large volumes and thereby eliminating the most proximal driver of passive overconsumption (see Figure 1). We therefore hypothesize such interventions to be more effective in reducing food intake than the ones that were evaluated in this research project. On the basis of the qualitative studies among point-of-purchase representatives, such reductions and eliminations seem risky for food companies and therefore not logical steps to take. In this light, formal legislation would be necessary, which leads to the question of the role of the government on this issue. As illustrated by both our focus group studies among consumers^24^ and former mayor Bloomberg's idle attempt to ban the larges sodas sizes in New York City,^44^ many people feel very strongly that governmental initiatives with respect to portion sizes are paternalistic. Our focus group data and the controversial ban of large soft drink size in NYC correspond to a vivid and wide-ranging debate about this issue.^45^ Apparently, people have negative attitudes towards perceived restrictions on their freedom of choice, especially if the government is involved. This is interesting to observe since it is, and always has been, a given fact that consumers are confronted with an assortment of food and available choices, offered and created by an external party. Thaler and Sunstein^46^ describe this as follows: ‘Just as no building lacks an architecture, so no choice lacks a context'. This implies that it is impossible to eliminate contextual factors that affect behavior, or as Quigley^47^ describes it: ‘Choice architecture is all around us. As such, our choices are constantly at the behest of a myriad of influences. We do not, therefore, even have to consider *new* policy initiatives to see that our choice-environment and health-affecting decisions are to some extent already shaped and constructed.'

Certainly, people should be held accountable for their own behavior. It is, however, questionable whether the food environment as it is currently designed, sufficiently facilitates consumers in making healthy choices. During the interviews, the point-of-purchase representatives claimed that consumers influence the assortment because demand creates the offerings and it is in the interest of the producers to respond to consumer demands. This seems (at least partly) true: the industry offers choices and consumers are free to choose that which and how much they eat. As illustrated by the same interviews with the point-of-purchase representatives;^24^ however, it was said by the participants that the main interest of the food industry is increasing its profits by selling as much food as possible. Therefore, in many cases, the goals of the food industry conflict with the interest of public health, often resulting in an unhealthy food environment. When applying this to portion sizes, the choice is often between large and extra large. Moreover, the industry actively creates a demand for large sizes, and, over time, larger sizes have been added to the portfolio while smaller sizes have been removed.^6,43^ This means that, as is the case with any given assortment, consumers' individual freedoms have been limited. Nevertheless, we found that consumers in our focus group study did not seem to consider restrictions to their freedom stemming from commercial parties (as opposed to governmental interventions) as paternalistic. This surprising and paradoxical observation leads us to describe this phenomenon as the ‘paternalization paradox'. As choice architecture is all around us anyway, Quigley^47^ argues however that there are reasons to prefer sets of health-affecting options that have been intentionally designed by the state (and are intended to contribute to the health of its citizens), rather than those that stem from, for instance, corporate parties (and rather directed at increasing profits than at people's health). Even so, possibly resulting from the paternalization paradox, governments seem unwilling to demand portion size reductions and the elimination of the largest portion sizes. Afraid of being accused of paternalism, governments prefer to stress the importance of people's individual responsibility and information provision.

An alternative approach to this dilemma seems to be ‘libertarian paternalism'. Libertarian paternalists try to steer people's behavior in welfare-promoting directions without blocking choices or eliminating freedom of choice.^46^ Libertarian paternalism is based on the assumption that people's choices and preferences are strongly influenced by contextual factors. One example of a contextual factor is that people do not tend to depart from the status quo situation. This means that default settings strongly steer people's choices even when better alternatives are available.^48^ Therefore, choice architects can design the choice environment and thereby ‘nudge' people to make behavioral choices that are in the best interests of their personal wellbeing and the public good.^49^ Instances of the use of nudges to affect behavior are organ donation or participation in cancer screening programs.^47^ According to libertarian paternalists, people should always be given the freedom to make their own perhaps unhealthy choices. However, unhealthy options should cease to be so heavily marketed or set as the default.

The principles of libertarian paternalism seem to be feasible and useful in shaping a more ‘portion-friendly' choice environment. Most importantly, this means that portion sizes that are in line with the nutritional guidelines should become the default option. This would mean that ‘healthy' portion sizes are the most visible and explicitly labeled and marketed. Furthermore, portion sizes as recommended by the nutritional guidelines should be the most attractively priced, and the sizes given unless otherwise requested. This means that the routinely asked question ‘Would you like that in small, medium, large or super-size?' would become superfluous and that customers will automatically receive the smallest item, unless they explicitly demand otherwise.

All in all, portion sizes have increased significantly over the past decades and can be considered an important obesogenic factor. The results of the research project described in this paper showed that portion size labeling, offering a larger variety of portion sizes, and proportional pricing were considered feasible to implement according to both consumers and point-of-purchase representatives. Studies into the effectiveness of these interventions demonstrated that the impact of portion size labeling on the (intended) consumption of soft drinks was, at most, modest. Furthermore, the introduction of smaller portion sizes of hot meals in worksite cafeterias in addition to the existing size stimulated a moderate number of consumers to replace their large meals by a small meal. Elaborating on these findings, we advocate further research into communication and marketing strategies related to portion size interventions; the development of both environmental portion size interventions and educational interventions that train people how to deal with a ‘super-sized' food environment; the implementation of regulation with respect to portion size labeling; and the use of nudges to stimulate consumers to select healthier portion sizes.
